# E-Cadherin Regulates Mitochondrial Membrane Potential in Cancer Cells

**DOI:** 10.3390/cancers13205054

**Published:** 2021-10-09

**Authors:** Hydari Masuma Begum, Chelsea Mariano, Hao Zhou, Keyue Shen

**Affiliations:** 1 Department of Biomedical Engineering, Viterbi School of Engineering, University of Southern California, Los Angeles, CA 90089, USA; hbegum@usc.edu (H.M.B.); cmariano@usc.edu (C.M.); zhouhao@usc.edu (H.Z.); 2 USC Stem Cell, Keck School of Medicine, University of Southern California, Los Angeles, CA 90033, USA; 3 Norris Comprehensive Cancer Center, Keck School of Medicine, University of Southern California, Los Angeles, CA 90033, USA

**Keywords:** mitochondrial membrane potential, tumor microenvironment, E-cadherin, adherens junction, MCF-7, MDA-MB-231, CRISPR/Cas9, breast cancer

## Abstract

**Simple Summary:**

Cancer cells have unusually high mitochondrial membrane potential (ΔΨ_m_). However, the microenvironmental mechanisms that regulate cancer cell ΔΨ_m_ remain unclear. In this study, we use in vitro micropatterned tumor models to mimic the confinement cues in tumor microenvironments and show that the E-cadherin mediated intercellular adhesion negatively regulates cancer cell ΔΨ_m_.

**Abstract:**

Epithelial cancer cells often have unusually higher mitochondrial membrane potential (ΔΨ_m_) than their normal counterparts, which has been associated with increased invasiveness in vitro and higher metastatic potential in vivo. However, the mechanisms by which ΔΨ_m_ in cancer cells is regulated in tumor microenvironment (TME) remain unclear. In this study, we used an in vitro micropatterning platform to recapitulate biophysical confinement cues in the TME and investigated the mechanisms by which these regulate cancer cell ΔΨ_m_. We found that micropatterning resulted in a spatial distribution of ΔΨ_m_, which correlated with the level of E-cadherin mediated intercellular adhesion. There was a stark contrast in the spatial distribution of ΔΨ_m_ in the micropattern of E-cadherin-negative breast cancer cells (MDA-MB-231) compared to that of the high E-cadherin expressing (MCF-7) cancer cells. Disruption and knockout of E-cadherin adhesions rescued the low ΔΨ_m_ found at the center of MCF-7 micropatterns with high E-cadherin expression, while E-cadherin overexpression in MDA-MB-231 and MCF-7 cells lowered their ΔΨ_m_ at the micropattern center. These results show that E-cadherin plays an important role in regulating the ΔΨ_m_ of cancer cells in the context of biophysical cues in TME.

## 1. Introduction

Epithelial cancer cells have higher mitochondrial membrane potential (ΔΨ_m_) than their normal counterpart cells [1], which has been associated with cancer stem cell features, increased secretion of angiogenic factor, and matrix metalloproteinase, as well as higher invasiveness in vitro [2,3,4,5]. We have previously reported in a xenograft metastatic breast cancer model in mice that cancer cells with higher ΔΨ_m_ result in a greater lung metastatic burden than those with low ΔΨ_m_ [6]. Together, these results highlight the biological significance of ΔΨ_m_ in cancer cells. However, the mechanisms by which it is differentially regulated in situ remain unclear.

The tumor microenvironment (TME) is a complex amalgamation of many types of cues, including different cell types such as fibroblasts and immune cells [7], biochemical cues from cellular metabolism/hypoxia and cell-type specific secretions or interactions [8,9,10], and physical cues such as solid stresses and matrix stiffness from tumor growth and extracellular matrix remodeling [11]. Among these, stromal cells have been found to fuel mitochondrial metabolism in cancer cells through metabolic coupling [12,13], while hypoxia-driven induction of transcription factors such as PGC1-α increases mitochondrial biogenesis in cancer cells [14]. Importantly, recent studies show an emerging role of mechanical cues from the TME such as ECM stiffness in influencing cancer cell metabolism through mechanotransduction, adhesion receptor signaling, and cytoskeletal reorganization [15]. We have lately reported a spatial distribution pattern of ΔΨ_m_ in cancer cells associated with physical confinement cues from the surrounding stromal cells using a micropatterning platform, the micropatterned tumor-stromal assay (μTSA) [6,16]. We showed that the physical confinement from TME downregulates ΔΨ_m_ in cancer cells, while the ΔΨ_m_ of those without confinement remains high [6]. Yet, the exact mechanisms by which the physical confinement regulates cancer cell ΔΨ_m_ remain to be determined.

In cancer cells, physical confinement has been found to induce changes in cell adhesion and cytoskeletal rearrangements, which alter their invasiveness and metastatic potential [17,18]. In particular, loss of E-cadherin, which forms a core component of intercellular adherens junctions (AJs) [19], is associated with increased migration and invasiveness in vitro and exacerbated lung metastases in vivo [20]. On the other hand, activating E-cadherin adhesions inhibits tumor metastases and decreases numbers of circulating tumor cells (CTCs) in the blood [21]. Lately, it was shown that E-cadherin plays a role in limiting oxidative stress and reactive oxygen species (ROS)-mediated apoptosis in cancer dissemination [22]. Whether E-cadherin regulates pathways directly affecting mitochondrial activity remains to be investigated, which could provide novel targets for cancer therapeutics.

In the present study, we investigated whether physical confinement cues can induce spatially regulated cell adhesion and how these, in turn, regulate ΔΨ_m_ level and its spatial distribution within TME. We show that pathways related to E-cadherin-mediated AJs are differentially regulated at the edge vs. center of the tumor model, and that E-cadherin expression correlates with ΔΨ_m_ spatial distribution. We further demonstrated that disrupting AJs rescues the ΔΨ_m_ level in confined cancer cells with lower ΔΨ_m_, while overexpressing E-cadherin decreases the ΔΨ_m_ level at the micropattern centers. Our work thus provides a novel insight into the potential role of E-cadherin mediated adhesions in regulating ΔΨ_m_ in cancer cells.

## 2. Materials and Methods

### 2.1. Cell Culture and Micropatterning

MCF-7 and MDA-MB-231 cells were obtained from American Type Culture Collection (ATCC) cultured as described previously [6] in Dulbecco’s Modified Eagle Medium (DMEM) with 10% Fetal Bovine Serum (FBS), 100 U/mL penicillin, and 100 μg/mL streptomycin (P/S). For the open edge unconfined micropatterns, an Epilog laser engraver was used to cut 2 mm diameter holes within a 10 mm diameter circular pattern in a 250 μm thick polydimethylsiloxane (PDMS) sheet. These PDMS stencils were aligned on collagen coated coverslips [6], treated with 0.2% Pluronic F-127 (Sigma-Aldrich, St. Louis, MO, USA), and rinsed with PBS prior to cell seeding. For cell seeding, 300,000 cancer cells were seeded per well in 1 mL of DMEM per well of a 24-well plate, with each well containing a PDMS stencil aligned onto a collagen coated coverslip. The well-plate was centrifuged at 200× *g* for 5 min, followed by the slowest deceleration setting. The cells were then incubated for 4–5 h, before micropatterns were rinsed with DMEM, placed in a fresh well with new DMEM, and incubated for 4 days prior to use in experiments. For confined cancer cell micropatterns, master molds (array of 500 μm diameter islands) were designed using AutoCAD (Autodesk, San Rafael, CA, USA) and fabricated through photolithography on silicon wafers using SU8 photoresist (100 μm thick). Sylgard 184 polydimethylsiloxane (PDMS, 10:1 base: curing agent) (Dow Corning, Midland, MI, USA) was poured on the wafers, degassed, and cured overnight at 65 °C, following which PDMS stamps were cut out using a 10 mm biopsy punch. Freshly mixed liquid PDMS (10:1 mix ratio) was spun coated onto 40 mm × 24 mm rectangular glass coverslips. These were incubated at room temperature for 45 min, then the PDMS stamps were dipped into the spun coated liquid PDMS and printed onto collagen coated coverslips. The coverslips were incubated overnight at room temperature to cure the PDMS, treated with 0.1% Pluronic, and rinsed with PBS prior to cell seeding (previously described seeding method; 300,000 MCF-7 cells per well). Confined cell micropatterns were cultured for 4 days to allow the cadherin-dominant micropatterns to form prior to experiments.

### 2.2. Generation of E-Cadherin-GFP Expressing and E-Cadherin Knockout Cell Lines

Plasmid DNA encoding E-cadherin-GFP was obtained from Addgene (plasmid # 28009 deposited by Jennifer Stow; http://n2t.net/addgene:28009 (accessed on 7 October 2021); RRID:Addgene_28009) [23]. Plasmid DNA was amplified with DH5α (Thermo Fisher, Waltham, MA, USA) and isolated using the QIAprep Spin Miniprep Kit (Qiagen, Hilden, Germany) according to manufacturer’s instructions, and the sequence was confirmed by Sanger sequencing with CMV-F, EGFP-N, and BGH-rev primers at GENEWIZ. A total of 200,000 MDA-MB-231 cells and 150,000 MCF-7 cells were seeded in 6-well plates and, after overnight incubation, transfected with E-cadherin-GFP plasmid DNA using the Effectene Transfection Reagent (Qiagen) according to manufacturer’s instructions (0.4 μg plasmid DNA per transfection). Cell culture media was changed 24 and 48 h post transfection, and cells were then passaged 1:5 in antibiotic selection media (DMEM, 10% FBS, 0.5 mg/mL geneticin, no P/S). Antibiotic selection was maintained until there were no cell colonies growing in the non-transfected control wells (7–10 days). Transfected cells were then expanded, and FACS sorted for GFP positive cells. Clustered regularly interspaced short palindromic repeats (CRISPR) technology was used to generate E-cadherin knockout (KO) MCF-7 cells. Briefly, 150,000 MCF-7 cells were seeded in a 6-well plate and allowed to adhere overnight. The next day, cells were transfected with 0.4 μg of E-cadherin CRISPR/Cas9 KO plasmids (sc-400031, which encode E-cadherin-specific 20 nt guide RNA sequences, SpCas9, and GFP reporter) using Effectene Transfection Reagent (Qiagen). Cell culture media was changed 24 h and 48 h post transfection. E-cadherin KO cells were then harvested, and FACS sorted by positive GFP fluorescence (transiently expressed by the transfected cells). Sorted KO cells were expanded for subsequent studies.

### 2.3. Mitochondrial Membrane Potential Staining and Imaging

Micropatterns were incubated in extracellular imaging buffer (130 mM sodium chloride, 5 mM potassium chloride, 1.5 mM calcium chloride, 1 mM magnesium chloride, 25 mM 4-(2-hydroxyethyl)-1-piperazineethanesulfonic acid (HEPES), 1 mg/mL BSA, and 5 mM glucose, with the pH adjusted to 7.4) with 10 nM tetramethylrhodamine methyl ester (TMRM, Life Technologies) for 45 min, and imaged in the same dye-containing buffer using a Nikon Eclipse Ti inverted microscope, using a Nikon Plan Fluor 10× objective with a numerical aperture (NA) of 0.30 (for unconfined micropatterns) or a Nikon Plan Apo 20× objective with 0.75 NA (for confined micropatterns). A Nikon C2 confocal microscope (Nikon Plan Apo 60× oil immersion objective, 1.40 NA) was used for confocal imaging.

### 2.4. Drug Treatment and Immunostaining

After 4 days of culture, micropatterns were treated with 1 mM or 10 mM 1,4-Dithiothreitol (DTT, MilliporeSigma, Burlington, MA, USA), live imaged for ΔΨ_m_ with TMRM (Life Technologies, Carlsbad, CA, USA) following which they were fixed in 4% paraformaldehyde (PFA) for 15 min, rinsed, and stored in PBS at 4 °C until they were immunostained. For antibody mediated inhibition of E-cadherin adhesions, unconfined MCF-7 micropatterns were treated at day 4 with 50 μg/mL anti-E-cadherin antibody (CD324 Monoclonal Antibody, clone DECMA-1, eBioscience^TM^ (ThermoFisher, Waltham, MA, USA) for 3 h in extracellular imaging buffer, and monitored for ΔΨ_m_ changes under 10 nM TMRM. For immunostaining, micropatterned cells on coverslips were permeabilized with 0.1% Triton X-100 for 10 min, and blocked in 4% bovine serum albumin (BSA) for 2 h. Samples were then incubated in primary antibody (anti-E-cadherin, clone 24E10, Cell Signaling, 1:200; anti-TOM20, sc-17764, Santa Cruz Biotechnology, 1:100) diluted in 4% BSA for 2 h. To validate CRISPR knockout of E-cadherin in MCF-7 cells, DECMA-1 (5 µg/mL) was used for E-cadherin immunostaining. Samples were then rinsed with PBS 3 times with 5 min for each rinse, before incubation in secondary antibody diluted in 4% BSA for 1 h. After the immunostaining, samples were further rinsed with PBS 3 times for 5 min per rinse, and mounted onto a glass slide using fluoro-gel II mounting medium (Electron Microscopy Sciences, Hatfield, PA, USA) before microscopic imaging.

### 2.5. Image Analysis and Quantification

Fluorescence images were analyzed with ImageJ (National Institutes of Health, NIH, Bethesda, MD, USA, last accessed on 7 October 2021). For radial distribution plots, a region of interest (ROI) was drawn around the micropatterned island, and a custom macro was used to extract pixel coordinates (normalized to the centroid) and pixel intensities within the selected ROI. A custom MATLAB (MathWorks, Portola Valley, CA, USA, last accessed on 7 October 2021) code was then used to convert the Cartesian coordinates to normalized polar coordinates, where the radial distances of 0 and 1 represent the center and edge of micropatterns, respectively. For region-based (center vs. edge) quantification of fluorescence, a custom ImageJ macro was used to select 5 ROIs each at these locations and calculate the average fluorescence (representative center and edge ROIs are shown in Figure 3a). The ITCN plugin on ImageJ was used for cell density quantifications. For the quantification of cell–cell contact index, outlines of individual cells were traced on ImageJ, and the index was calculated by dividing the overlapped area (AND) by the total combined contour area (OR).

### 2.6. Statistical Analysis

Data were presented as the mean ± S.D. (standard deviation). All statistical analyses (unpaired *t*-tests, one-way ANOVA, and 2-way ANOVA) were performed on GraphPad Prism, and the resulting *p*-values are indicated for each figure. N.s.: not significant (*p* > 0.05), *: *p* < 0.05, **: *p* < 0.01, ***: *p* < 0.001, and ****: *p* < 0.0001.

## 3. Results

### 3.1. Adherens Junctions (AJs) Are Downregulated at the Tumor-stromal Interface in a Micropatterned Tumor Model

**Figure 1:**
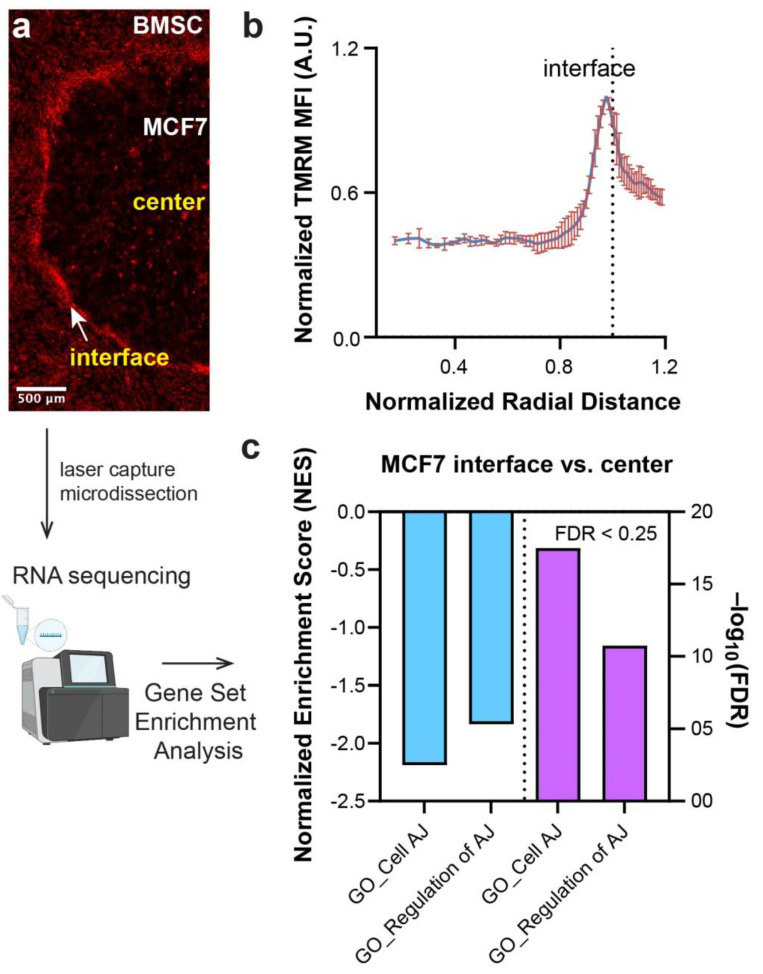
Spatial distribution of ΔΨ_m_ of MCF-7 cells in micropatterned tumor model associated with regulation of cell adhesion. (**a**) Representative image showing TMRM fluorescence of a day 4 MCF-7-BMSC co-culture micropattern and (**b**) the corresponding normalized radial distribution. (**c**) Gene set enrichment analysis of MCF-7 cells at the tumor-stromal interface relative to MCF-7 cells at the center of the tumor island, following RNA-sequencing of laser capture microdissected from different locations of the micropattern as described in [6] with a false discovery rate (FDR) < 0.25.

We have previously established a micropatterning platform, the micropatterned tumor-stromal assay (μTSA), to recapitulate tumor-stromal interactions [16], and demonstrated a spatial gradient of ΔΨ_m_ in MCF-7 breast cancer cells within the tumor island surrounded by bone marrow stromal cells (BMSCs) [6]. Within µTSA, MCF-7 cells in regions near the center of the micropattern had lower ΔΨ_m_ (visualized by the red TMRM staining) than those closer to the tumor-stromal interface (Figure 1a). Quantitative analysis showed a close to 3-fold difference in ΔΨ_m_ level between the two regions (Figure 1b). We extracted MCF-7 cells from the center and interface of the tumor islands using laser capture microdissection (LCM), and performed RNA sequencing to examine the differential regulation of gene expression between the two regions [6]. Gene set enrichment analysis (GSEA) [24,25] revealed a significant negative enrichment of pathways related to adherens junctions (AJs) in MCF-7 cells at the interface relative to the center (Figure 1c), suggesting a spatial distribution of differential cell adhesions (mediated by AJs) within the tumor island that negatively correlates with ΔΨ_m_ spatial distribution. As confinement cues were shown to induce changes in cancer cell adhesion in vitro [18], we hypothesized that the physical confinement cues induce ΔΨ_m_ changes by regulating the level of AJs in cancer cell adhesion.

### 3.2. E-Cadherin Expression Correlates with Spatial Distribution of ΔΨ_m_ within Tumor Micropattern

**Figure 2:**
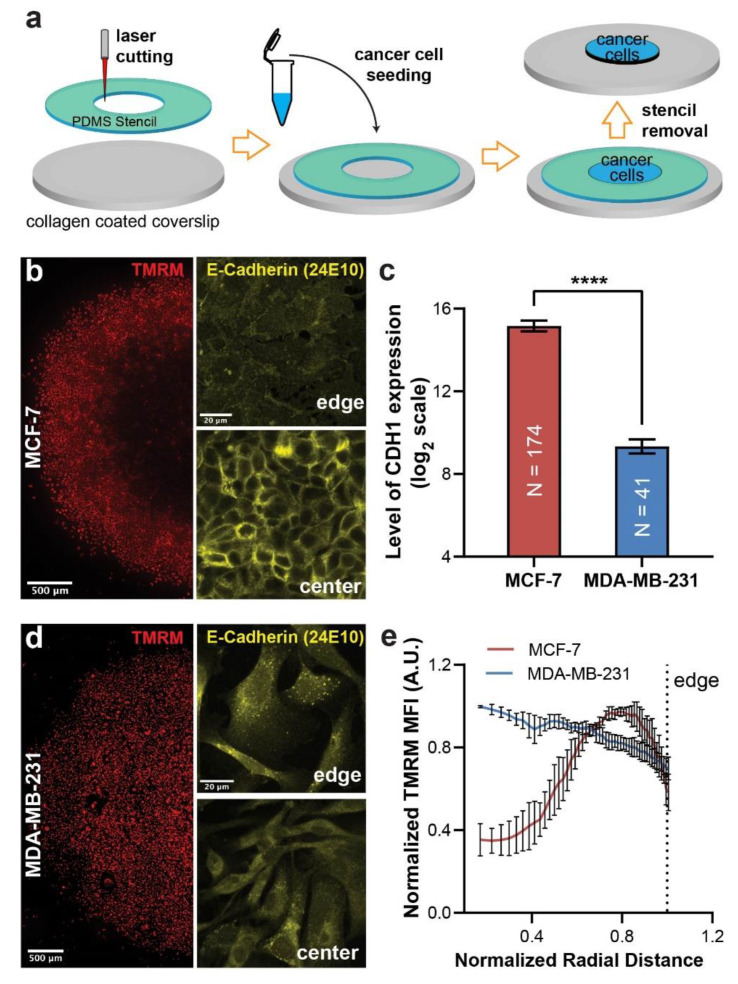
Correlation of E-cadherin expression with spatial distribution of ΔΨ_m_. (**a**) Schematics of creating an unconfined monoculture micropattern. (**b**) Widefield low-magnification imaging showing spatial distribution of TMRM fluorescence and high-magnification confocal imaging showing E-cadherin localization in day 4 MCF-7 monoculture micropatterns. (**c**) Average E-cadherin (CDH1) expression in MCF-7 cells compared to MDA-MB-231 cells from > 40 independent experiments (obtained from Genevestigator database [27]). **** *p* < 0.0001 in an unpaired *t*-test. (**d**) Spatial distribution of TMRM fluorescence and confocal images of E-cadherin staining in day 4 MDA-MB-231 micropatterns. (**e**) Normalized radial distributions of TMRM fluorescence in day 4 MCF-7 and MDA-MB-231 micropatterns (3 micropatterns per condition).

To eliminate the impact of tumor-stromal biochemical signaling [16], we created a micropatterned monoculture of MCF-7 cells on collagen coated coverslips (Figure 2a). After 4 days of culture, MCF-7 cells also formed a spatial pattern of ΔΨ_m_ distribution with low ΔΨ_m_ in the center and high ΔΨ_m_ at the edge (Figure 2b), although the area of cells with higher ΔΨ_m_ was greater than those in the co-cultured micropatterns (Figure 1a). As the MCF-7 monoculture micropatterns retained the center-edge spatial ΔΨ_m_ gradient, we used this model and its fully confined variant to assess the role of spatial confinement and cell–cell adhesion in regulating ΔΨ_m_ levels for the rest of the study. We first examined whether there was a differential pattern of AJ formation within the micropatterns. We immunostained the micropatterns against E-cadherin, a core component of AJs in epithelial cells [19]. Confocal imaging revealed that MCF-7 cells at the edges of the micropattern had lower E-cadherin expression with cytoplasmic localization (Figure 2b). In contrast, MCF-7 cells at the center of micropatterns showed higher E-cadherin expression with distinct cell membrane localization, forming a cobblestone-like structure characteristic of epithelial monolayer that is mediated by AJ formation [26].

To examine the role of E-cadherin in regulating spatial distribution of ΔΨ_m_ within the micropatterns, we next picked a breast cancer cell line that has lower/negative E-cadherin expression, to test if they form a different ΔΨ_m_ pattern. The Genevestigator database [27] shows that MDA-MB-231, a metastatic breast cancer cell line, has significantly lower E-cadherin expression than MCF-7 cells in data collected from > 40 independent experiments (Figure 2c). When MDA-MB-231 cells were micropatterned and live imaged for ΔΨ_m_, they demonstrated a spatial distribution of ΔΨ_m_ levels distinct from that in MCF-7 micropattern, where the ΔΨ_m_ of MDA-MB-231 cells was similar/slightly higher at the center than those at the edge of the micropatterns (Figure 2d,e). E-cadherin immunostaining and confocal imaging of MDA-MB-231 cells in the micropattern confirmed that E-cadherin expression in these cells was essentially absent at the cell membrane, and displayed similar intracellular characteristics between cells at the edge and center of the micropattern (Figure 2c). Together, these results suggested a potential role of E-cadherin-mediated AJ formation in regulating ΔΨ_m_ in cancer cells.

### 3.3. Disrupting AJ Formation Increases ΔΨ_m_ in MCF-7 Micropattern

**Figure 3:**
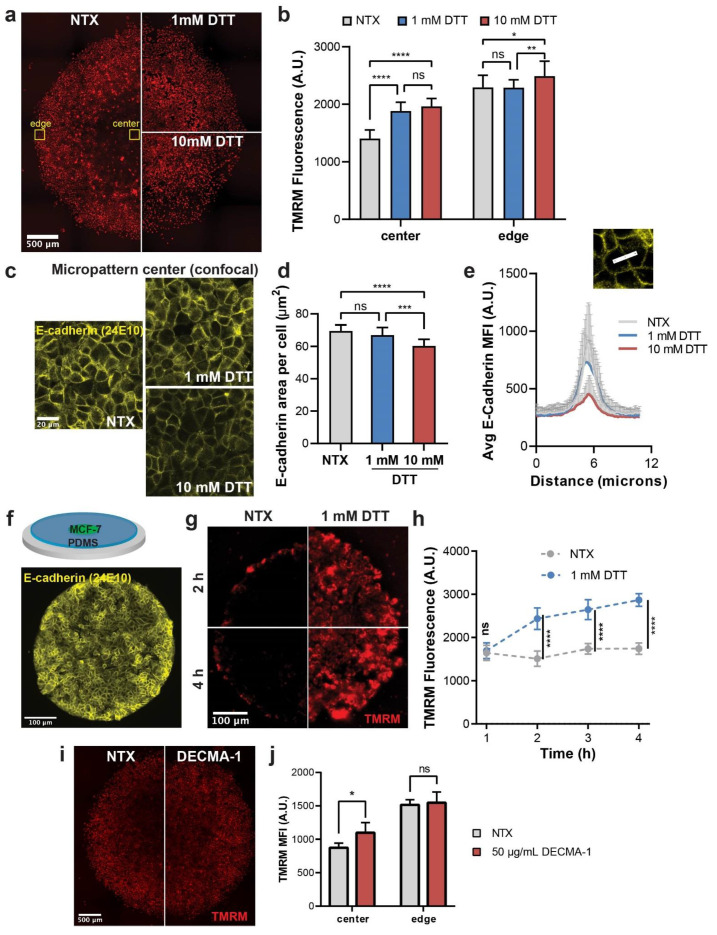
Disruption of AJs with DTT in MCF-7 micropatterns. (**a**) TMRM fluorescence of day 4 MCF-7 unconfined micropatterns with and without 1 mM and 10 mM DTT treatment (3 h). (**b**) Quantification of average TMRM fluorescence at the centers and edges of the micropatterns shown in (**a**). * *p* < 0.0332, ** *p* < 0.0021, and **** *p* < 0.0001 in a 2-way ANOVA. (**c**) Confocal imaging showing E-cadherin fluorescence at the centers of day 4 MCF-7 unconfined micropatterns with and without indicated DTT treatment. (**d**) Average E-cadherin area per cell in MCF-7 cells shown in (**c**). *** *p* < 0.0002 and **** *p* < 0.0001 in an ordinary one-way ANOVA. (**e**) Line scans showing average E-cadherin fluorescence across intercellular cadherin adhesions as shown in schematic. At least 18 cell pairs were analyzed per condition. (**f**) E-cadherin staining showing AJ formation in an MCF-7 micropattern confined with a thin layer of PDMS. (**g**) TMRM fluorescence of MCF-7 cells in confined micropatterns before and after 2 h and 4 h of 1 mM DTT treatment. (**h**) Quantification of TMRM fluorescence in MCF-7 confined micropatterns treated with 1 mM DTT over 4 h. **** *p* < 0.0001 in an ordinary one-way ANOVA. (**i**) TMRM fluorescence of MCF-7 cells in unconfined micropatterns without or with 50 μg/mL anti-E-cadherin (DECMA-1) treatment for 3 h. (**j**) Quantification of ΔΨ_m_ at the center and edge of micropatterns shown in (**i**). ns: not significant (*p* > 0.05); * *p* < 0.05 in a 2-way ANOVA.

We next aimed to investigate the effect of disrupting E-cadherin mediated AJs on the spatial distribution of ΔΨ_m_ in MCF-7 micropatterns. We used 1,4-dithiothreitol (DTT), a reducing agent that disrupts E-cadherin mediated cell–cell adhesion by cleaving the disulfide bonds in the extracellular domains of E-cadherin [28]. At a concentration of 10 mM, DTT has been shown to selectively disrupt AJs in MDCK cells [29]. We treated MCF-7 micropatterns at day 4 with 1 mM and 10 mM DTT, and observed a significant increase in ΔΨ_m_ in MCF-7 cells at the centers of the micropatterns compared to the untreated control (Figure 3a,b). On the other hand, in MCF-7 cells at the edges of the micropattern, only the higher DTT concentration (10 mM) led to a significant increase in ΔΨ_m_. Confocal imaging of E-cadherin immunostaining in MCF-7 cells revealed that the 10 mM DTT treatment significantly decreases the E-cadherin level per cell at the center of the micropattern (Figure 3c,d). Moreover, we saw a dose-dependent decrease in fluorescence intensity in E-cadherin at intercellular junctions with DTT treatment, with 10 mM showing a more marked decrease than the 1 mM DTT treatment (Figure 3e). Interestingly, we noticed that, while the lower DTT concentration (1 mM) did not significantly reduce AJ area (Figure 3d), it was sufficient to increase ΔΨ_m_ in MCF-7 cells at the micropattern center. We thus tested the response time of ΔΨ_m_ to the DTT treatment using the 1 mM DTT concentration. We created a confined micropattern of MCF-7 cells with a thin surrounding layer of PDMS (Figure 3f). After 4 days of culture, MCF-7 cells formed a cadherin-dominant micropattern with uniformly high E-cadherin level at cell–cell junctions throughout the tumor island (Figure 3f). As expected, the ΔΨ_m_ of the MCF-7 cells in the micropattern became very low (Figure 3g), which was similar to that at the center of the open edge micropatterns. Upon treatment with 1 mM DTT, we observed a significant increase in the ΔΨ_m_ level as soon as after 2 h into the treatment (Figure 3g,h). To further validate the impact of disrupting E-cadherin mediated AJ formation/cell–cell adhesion, we treated MCF-7 micropatterns with a function-blocking E-cadherin monoclonal antibody, DECMA-1, which has been reported to disrupt E-cadherin mediated AJs in MCF-7 cells [30] (Figure 3i). Similar to the DTT treatment, DECMA-1 treatment significantly increased ΔΨ_m_ of cancer cells at the center, but not at the edge of unconfined micropatterns (Figure 3i,j). These results suggest that the AJ formation by E-cadherin in cancer cells negatively regulates the ΔΨ_m_ level in MCF-7 cancer cells.

### 3.4. E-Cadherin Expression in MDA-MB-231 Cells Decreases ΔΨ_m_ at the Micropattern Center

**Figure 4:**
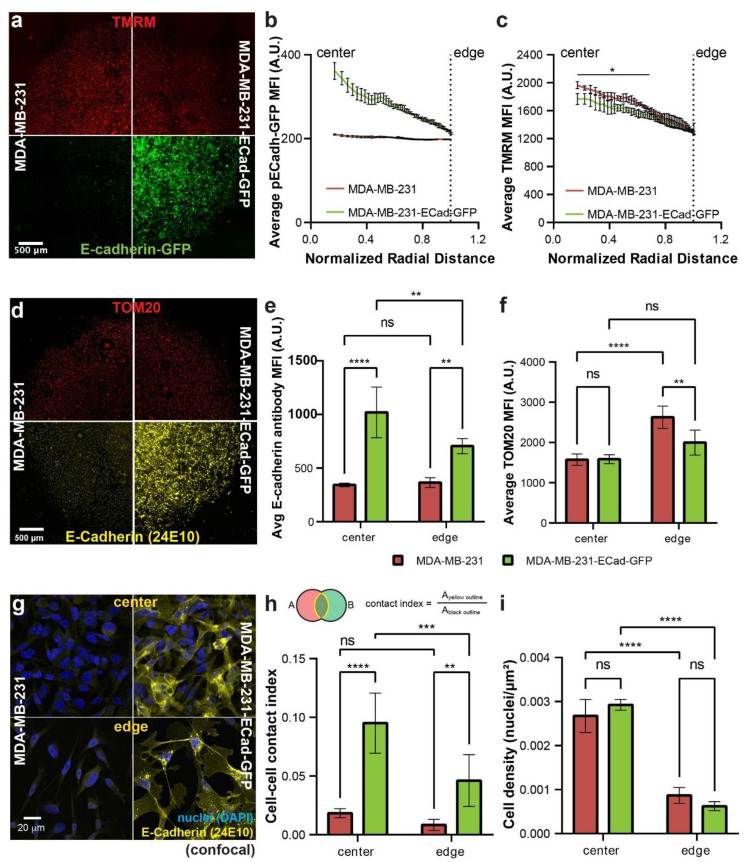
Effect of E-cadherin expression on the spatial distribution of ΔΨ_m_ in MDA-MB-231 micropatterns. (**a**) TMRM and E-cadherin-GFP fluorescence of day 4 unconfined micropatterns of MDA-MB-231 (non-transfected control) and MDA-MB-231 cells transfected with E-cadherin-GFP (widefield imaging). (**b**) Radial distribution of E-cadherin-GFP in E-cadherin-GFP transfected vs. non-transfected micropatterns as shown in (**a**), *n* = 4 micropatterns per condition. (**c**) Radial distribution of ΔΨ_m_ of representative micropatterns shown in (**a**), *n* = 4 micropatterns per condition. (**d**) Immunofluorescence widefield imaging showing TOM20 and E-cadherin antibody fluorescence in E-cadherin-GFP transfected vs. non-transfected controls. Quantification of mean fluorescence intensities of (**e**) E-cadherin and (**f**) TOM20 immunostaining at the centers and edges of the immunostained micropatterns shown in (**d**). (**g**) Confocal imaging showing E-cadherin fluorescence at the centers and edges of MDA-MB-231 and MDA-MB-231-ECadherin-GFP micropatterns. Quantification of (**h**) cell–cell contact index and (**i**) cell density at the centers and edges of the micropatterns. Contact index defined as the ratio of cell–cell overlap area and total area of the contour. For (**b**), all data points on the two curves are statistically different from each other at each radius (*p* < 0.05 by *t*-test); for (**c**), data points under the solid line are statistically different from each other at each radius (*p* < 0.05 by *t*-test); for (**e**,**f**,**h**,**i**): ** *p* < 0.0021, *** *p* < 0.0002, and **** *p* < 0.0001 in a 2-way ANOVA.

We further examined whether re-expression of E-cadherin in MDA-MB-231 cells, which have low/no E-cadherin expression, would induce a spatial regulation of ΔΨ_m_ levels in the micropattern. We transfected MDA-MB-231 cells with an E-cadherin-GFP construct [23], and created open-edge micropatterns with these cells alongside the wild-type (WT) MDA-MB-231 cells as control (Figure 4a, bottom). We confirmed the expression of E-cadherin by observing the E-Cadherin-GFP signal in the micropattern, which was higher at the center than the edge (Figure 4b). We also monitored the spatial distribution of ΔΨ_m_ in the micropatterns with TMRM live staining (Figure 4a, top). ΔΨ_m_ at the center of micropattern with MDA-MB-231 cells expressing E-cadherin was lower than that with WT MDA-MB-231 cells. Although we did not observe a similar edge vs. center pattern of the ΔΨ_m_ levels in MDA-MB-231-Ecad-GFP cells as with MCF-7 cells, the region with downregulated ΔΨ_m_ levels (vs. control cells) correlated with the elevated E-cadherin-GFP signal at the center of micropattern (Figure 4b,c). The expression of E-cadherin in transfected MDA-MB-231 cells and the center-edge difference was further confirmed with immunostaining and regional quantification in micropatterns (Figure 4d, bottom, and e). We also assessed whether the decrease in ΔΨ_m_ at the micropattern center in the E-cadherin expressing cells was due to a decrease in mitochondrial mass. Immunostaining of these micropatterns against TOM20, a mitochondrial protein indicative of mitochondrial mass [6], revealed that there was no difference in mitochondrial mass at the centers of these micropatterns (Figure 4d,f). Interestingly, there was significantly lower mitochondrial mass at the edge of micropattern with MDA-MB-231-Ecad-GFP cells, where no ΔΨ_m_ difference was observed, further supporting the notion that mitochondrial mass did not contribute to the ΔΨ_m_ differences.

Our RNA-seq and inhibition experiments pointed to the importance of E-cadherin mediated cell–cell adhesion (AJs) in ΔΨ_m_ regulation. Under high-magnification confocal microscopy, although we did not observe a completely epithelial morphology in the E-cadherin expressing MDA-MB-231 cells, these cells did exhibit morphological changes and increased cell–cell contact through overlaps of cell protrusions and/or cell bodies (Figure 4g). We defined a cell–cell contact index as the ratio of the overlapped area between two adjacent cells over the total contour area of the two cells (Figure 4h). We found that there was significantly higher cell–cell overlap/contact in micropatterns with E-cadherin expressing cells than WT MDA-MB-231 cells both at the center and edge of micropattern, whereas the difference was more significant at the center than the edge (Figure 4h). We also ruled out the possibility that such changes were caused by differences in cell density (Figure 4i). Together, these results indicate that re-expression of E-cadherin in MDA-MB-231 cells lowers the ΔΨ_m_ at micropatterns center through E-cadherin mediated cell–cell adhesion.

### 3.5. E-Cadherin Knockout and Overexpression Alter ΔΨ_m_ at the Center of MCF-7 Micropattern

**Figure 5:**
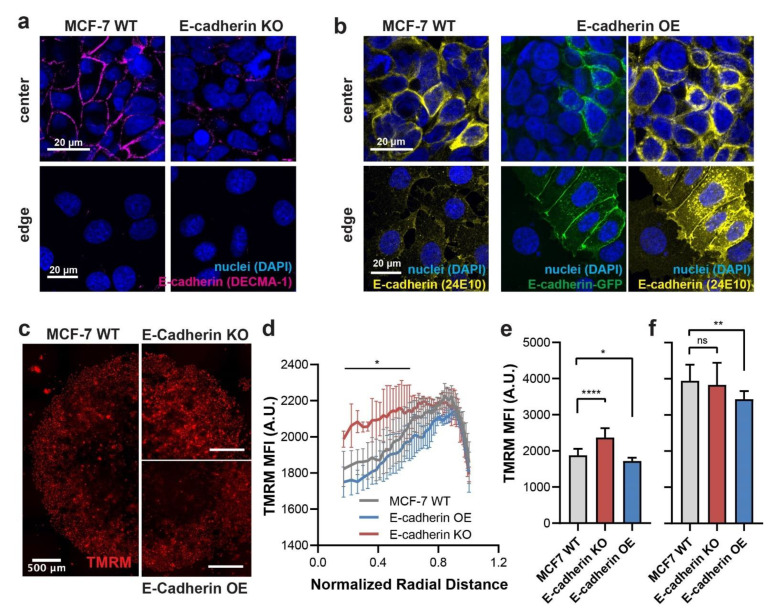
Effects of E-cadherin knockout (KO) and overexpression (OE) on ΔΨ_m_ in MCF-7 micropatterns. (**a**) Confocal imaging showing the localization and level of extracellular E-cadherin (immunostained by DECMA-1) at the centers and edges of unconfined micropatterns formed by WT and E-cadherin KO MCF-7 cells on day 4. (**b**) Confocal imaging showing the localization and level of E-cadherin by GFP signal and immunostaining (24E10) at the centers and edges of micropatterns formed by WT and E-cadherin OE MCF-7 cells on day 4. (**c**) Spatial distribution of ΔΨ_m_ indicated by TMRM fluorescence in unconfined micropatterns of WT, E-cadherin KO, and E-cadherin OE MCF-7 cells on day 4. (**d**) Radial distribution of ΔΨ_m_ in micropatterns shown in (**c**). Data points under the solid line are statistically higher in E-cadherin KO cells compared to WT cells at each radius (*p* < 0.05 by 2-way ANOVA). Quantification of average TMRM fluorescence at the centers (**e**) and edges (**f**) of micropatterns shown in (**c**). * *p* < 0.05, ** *p* < 0.01, and **** *p* < 0.0001 in an ordinary one-way ANOVA; *n* = 3 micropatterns per condition.

MCF-7 cells express high levels of E-cadherin compared to MDA-MB-231 cells (Figure 2c). We next investigated whether knocking out or further overexpressing E-cadherin could affect the ΔΨ_m_ of MCF-7 cells within the micropatterns. To create E-cadherin knockout (KO) cells, we transfected MCF-7 cells with a commercial E-cadherin CRISPR/Cas9 knockout kit that contains three plasmids, each encoding Cas9 and a specific guide RNA sequence against CDH1 (E-cadherin) site in the genomic DNA. To validate the knockout, we immunostained the transfected MCF-7 cells in unconfined micropatterns with the DECMA-1 E-cadherin antibody on day 4 (Figure 5a). We observed a marked decrease in E-cadherin immunostaining at the intercellular junctions in the center of micropatterns formed by the E-cadherin KO cells compared to the WT MCF-7 cells (Figure 5a, top panels). At the micropattern edges, both WT and KO cells expressed minimal E-cadherin at the cell–cell borders (Figure 5a, lower panels), similar to what was observed in immunostaining with 24E10 E-cadherin antibody in micropatterns of WT cells (Figure 2b).

To create E-cadherin overexpressing (OE) cells, we transfected MCF-7 cells with the same E-cadherin-GFP construct [23] used for MDA-MB-231 cells in Figure 4. We confirmed the overexpression of E-cadherin by both GFP fluorescence and immunostaining (Figure 5b). Notably, we saw a fraction of WT MCF-7 cells in the E-cadherin OE micropatterns due to incomplete killing of WT MCF-7 cells in antibiotic selection (Figure 5b, DAPI+GFP- cells). While both WT and E-cadherin OE cells had high expression of E-cadherin in micropattern center, the E-cadherin OE cells (GFP+) had visibly higher E-cadherin immunostaining than the WT (GFP-) cells. Importantly, at the micropattern edge, the E-cadherin OE cells demonstrated AJ formation indicated by the presence of E-cadherin-GFP and immunostaining signals at the cell–cell boundary. In contrast, those WT (GFP-) cells had negligible E-cadherin immunostaining in the same region (Figure 5b, lower panels).

We then measured the differences of ΔΨ_m_ spatial distribution in unconfined micropatterns with WT, E-cadherin KO, and E-cadherin OE MCF-7 cells. Upon live-staining of ΔΨ_m_ with TMRM, we found that the E-cadherin KO cells had significantly higher ΔΨ_m_ than the WT cells at the micropattern center without affecting those at the edge (Figure 5c–f). In contrast, the E-cadherin overexpression further significantly reduced ΔΨ_m_ at the micropattern center when compared to WT cells (Figure 5c–f). Interestingly, E-cadherin overexpression resulted in an overall decrease in ΔΨ_m_ at the micropattern edge (Figure 5f), which is consistent with the observation of AJ formation by the E-cadherin OE cells in this region (Figure 5b). These results further reinforce the essential role of E-cadherin in negatively regulating ΔΨ_m_ of cancer cell in our micropatterned tumor model.

## 4. Discussion

Loss of E-cadherin is widely known as an important step in the metastatic cascade; cancer cells that undergo the epithelial-mesenchymal transition (EMT) lose E-cadherin, allowing them to reduce intercellular adhesions and break off from the primary tumor [20]. On the other hand, after tumor dissemination, the loss of E-cadherin is also associated with increased oxidative stress and poor proliferation in the in vitro organoid tumor models [22]. However, it is unclear whether E-cadherin loss induces oxidative stress or if the accumulation of oxidative stress potentiates the loss of E-cadherin. Studies have shown that introduction of oxidative stress via H_2_O_2_ treatment leads to disruption of E-cadherin mediated AJs in MCF-7 breast cancer cells and overall reduction in E-cadherin expression in hepatocellular carcinoma cells [31,32]. On the other hand, overexpression of E-cadherin in gastric cancer cells led to enhanced mitochondrial and glycolytic metabolism [33]. Fragments of a different type of cadherin adhesion molecule called Fat (Ft) cadherin have been found to directly bind to complexes of the mitochondrial electron transport chain (ETC) and stimulate mitochondrial metabolism in *Drosophila* [34]. However, a mechanistic understanding of whether and how E-cadherin regulates mitochondrial activity in cancer cells remains lacking. In this study, we have shown that E-cadherin expression and in particular E-cadherin mediated AJ formation negatively regulates ΔΨ_m_ in cancer cells. The present study highlights a novel pathway wherein confinement cues from the TME regulate the ΔΨ_m_. Further studies are needed to investigate the mechanisms and molecular adaptors by which E-cadherin expression could regulate ΔΨ_m_, and its functional implications on cancer cell behavior.

## 5. Conclusions

In conclusion, we identified a novel mechanism of negative regulation of cancer cell ΔΨ_m_ by the E-cadherin mediated intercellular adhesion, the latter of which is upregulated by physical confinements in the tumor microenvironment. Our findings thus provide new insights into the roles of both extrinsic (tumor microenvironment) and intrinsic (adhesion molecule) cues in tumor progression.

## Data Availability

All data will be made available from the corresponding author upon reasonable request.
